# The impact of patient information on prescribing errors: Insights from pharmaceutical interventions

**DOI:** 10.1016/j.rcsop.2025.100665

**Published:** 2025-10-06

**Authors:** Daisuke Koike, Masahiro Ito, Keiko Tomomatsu, Ryuta Shindo, Terumi Miyashita, Junichi Yamakami, Akihiko Horiguchi, Shigeki Yamada

**Affiliations:** a Department of Gastroenterological Surgery, Fujita Health University School of Medicine, Bantane Hospital, 3-6-10, Otobashi, Nakagawa ward, Nagoya Zip cord: 454-8509, Japan; b Department of Quality and Safety in Healthcare, Fujita Health University Hospital, Toyoake, 1-98, Dengakugakubo, Kutsukakecho, Toyoake, Aichi Zip cord: 470-1192, Japan; c Department of Pharmacotherapeutics and Informatics, Fujita Health University School of Medicine, Toyoake, 1-98, Dengakugakubo, Kutsukakecho, Toyoake, Aichi Zip cord: 470-1192, Japan

**Keywords:** Medication error, Patient safety, Pharmaceutical intervention, Computerized physician order entry, Clinical decision support system

## Abstract

**Background:**

Medication errors are more likely to occur in patients with complex conditions, where appropriate prescribing requires accurate and comprehensive patient information. Inadequate use of such information, such as overlooking laboratory results or patient weight, can lead to dosing errors or contraindicated prescriptions, even with electronic checking systems. This study aimed to analyze prescribing errors detected through pharmaceutical interventions, focusing on the patient information in the hospital information system.

**Methods:**

A retrospective analysis was conducted on 9774 pharmaceutical interventions performed between April 2019 and March 2020. Of these, 3372 interventions related to patient information stored in hospital information system were included. Prescribing errors were categorized into five patient-specific information categories: allergy information, laboratory data, concomitant drugs, patient weight, and patient status and history. Demographic and prescription data were analyzed, and a heatmap was developed to visualize high-risk areas.

**Results:**

Among the included interventions, 1352 (40.1 %) prescriptions involved corrections of prescribing errors, with laboratory data being the most frequently utilized patient information source (*n* = 2526). Error rates were higher in weekend settings compared to weekday settings (56.2 % vs. 38.3 %, *P* < 0.001, Cramér's V = 0.111), and prescriptions for patients younger than 20 years exhibited the highest error rates among age groups (66.9 %; *P* < 0.001). Error rates were significantly varied by drug type (P < 0.001, Cramér's V = 0.395). Among these, digestive drugs showed the highest error rates (69.1 %), particularly those requiring renal dosing adjustments. Conversely, anti-tumor agents demonstrated a lower error rate (15.9 %) due to stringent monitoring processes. The high-risk heatmap highlighted specific risks, such as weight data for younger patients and patient status for anti-inflammatory drugs.

**Conclusion:**

Laboratory data were the most frequently used information source, to prevent prescribing errors. The risk heatmap demonstrated weekends, pediatric patients, and renal dosing as high-risk areas. These findings suggest that future information systems should enhance the utility of laboratory data and incorporate tailored alerting strategies focused on high-risk patient conditions and clinical settings, such as real-time lab data alerts or weight-based dosing calculators, and potentially explore the use of AI for proactive error prevention.

## Highlights

• Pharmaceutical interventions can detect flaws in information-check systems.
• One-third of interventions find potential risks bypassing information-check systems.
• The most missed information in prescribing process was patient laboratory data.
• High-risk areas included weekends, pediatric patients, and renal dosing.

## Graphical abstract

**Figure:**
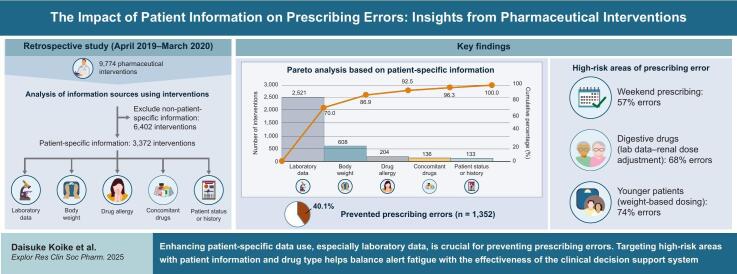

## 1 Introduction

Medication errors pose a critical challenge in healthcare in worldwide, affecting millions of patients each year and, in some cases, resulting in severe outcomes, including catastrophic harm.^1^, ^2^, ^3^, ^4^, 5. The medication delivery process is divided into several stages: physician prescribing, pharmacist verification, dispensing, administration and monitoring. Among these, the prescribing stage is where the highest number of medication errors occur.^5^ This makes it a critical point for intervention to reduce errors and improve patient safety. To prevent prescribing errors, there are two main areas that require alerting based on the nature of the information: one focuses on the prescription itself, such as dosage and usage restrictions, and another checks the prescribing drugs against patient-specific information, such as drug-drug or drug-allergy interactions, renal function, and conditions like pregnancy.^6^, ^7^, ^8^, ^9^ Electronic information-checking systems, commonly known as Computerized Physician Order Entry systems and Clinical Decision Support systems, have been implemented to address prescribing errors.^10^, ^11^, 12., 13. Despite these efforts, many studies have reported higher rates of medication errors in complex clinical environments where managing a large amount of patient-specific information is critical. For example, patients admitted in intensive care units or those with complex comorbidities, as well as elderly and pediatrics patients, have been shown to be at higher risk for prescribing errors.^14^, ^15^, ^16^ Weight-based dosing errors are also frequently reported in pediatric prescribing.^17^^,^^18^ Many patients with chronic kidney disease have suffered from renally inappropriate medication prescriptions,^19^^,^^20^ and liver function test are also critical patient specific data for appropriate prescribing.^21^ Additionally, drug-drug and drug-chemotherapy interactions were also commonly overlooked in malignant patients; approximately two-thirds of these patients were prescribed potentially inappropriate medications, and in 5.4 % of cases these errors impaired the chemotherapy process.^22^^,^^23^ These findings suggest that the complexity of patient conditions increases the risk of oversight. Without adequate integration of patient-specific and drug safety information, including dosing and interactions, critical steps may be missed, directly leading to prescribing errors.

High override rates of alerts have also been evident alongside alert fatigue, meaning that alerts had little impact on improving physicians' prescribing processes.^11^^,^^24^, ^25^, ^26^, ^27^ A recent review showed significant heterogeneity in data utilization among systems.^7^^,^^8^ However, little is known about which types of patient information in these systems are most critical for preventing prescribing errors. Further research is required to explore how patient-specific information influences prescribing errors and to identify high-risk areas where interventions can be most effective.

Pharmaceutical interventions are often performed during the pharmacist verification process to detect prescription errors that bypass the physician's prescribing phase and the electronic checking system, and their efficacy has been established.^28^, ^29^, ^30^, ^31^, ^32^ On the other hand, these interventions also highlight the limitations of current information-checking systems. This study aimed to investigate the relationship between prescribing errors related to patient-specific information and to identify high-risk areas of prescription errors by analyzing the outcomes of pharmaceutical interventions.

## 2 Method

### 2.1 Study setting and materials

This study retrospectively analyzed data collected from a tertiary academic hospital in Japan, where an electronic prescribing system has been implemented in hospital information system (HIS). The prescribing process was managed as follows: physicians prescribed medications based on the patient's medical condition, with all prescriptions ordered through the HIS. This system includes an alert function that checks for dosage and usage restriction, allergies and drug-drug interactions with concomitant drugs. After the physician's order, clinical pharmacists verified the prescriptions. In Japan, pharmacists are required to intervene by contacting the prescriber whenever discrepancies, potential errors, or unclear instructions are identified. This process is aimed at ensuring patient safety and proper medication use. If there were any concerns about the appropriateness or suitability of a prescription for the patient's condition, pharmacists would raise pharmaceutical interventions to clarify potential prescribing errors. All interventions were logged using a standardized template at the time of occurrence. Once verified, the medications were prepared and dispensed to the patients.

We retrospectively extracted data from the intervention report of the pharmaceutical department registry between April 2019 and March 2020. The interventions were recorded based on total drug prescriptions rather than lines in drug prescriptions. The registry data included patient age, sex, date, department, reason for inquiry, and inquiry outcome. Since the study aimed to explore the relationship between patient-specific information in the HIS and prescribing errors, the information sources for reasons for interventions were classified into five categories based on how they were organized in the HIS: allergy information, laboratory data, concomitant drugs, patient weight, and patient status and history. Patient status and history included conditions such as asthma, hemodialysis (HD), diabetes, and pregnancy. Prescribing errors were defined as prescriptions that required correction through pharmaceutical interventions. Pharmaceutical interventions concerning future prescriptions, including dosage or scheduling recommendations and pre-prescribing discussions with physicians, were not included in this study.

### 2.2 Alert setting for patient information source

Alerts varied based on the type of patient information. Information such as patient weight, laboratory data, and patient status and history were not automatically flagged during the prescribing by physicians, unlike allergy information and concomitant drugs. Physicians were required to manually check this data on separate pages within the HIS. Allergy alerts were triggered if a drug allergy had been registered using drug codes in HIS and if the prescribed drug met predefined conditions. Similarly, drug-drug interaction alerts appeared on the ordering screen when potential interactions were detected. For pharmacists, the system automatically provided information for patient laboratory data and all registered allergy information when processing prescription orders.

### 2.3 Statistical and visual analysis

Statistical analysis was performed using EZR (Saitama Medical Center, Jichi Medical University, Saitama, Japan; https://www.jichi.ac.jp/saitama-sct/SaitamaHP.files/statmedEN.html). Numerical data were expressed as mean with standard deviations, or as counts and percentage. Chi-square and Fisher's exact tests were used to assess associations between categorical variables. Fisher's exact test was used when the sample size or expected count was less than 5 to avoid inaccuracies associated with the chi-square approximation. When exact calculation was computationally infeasible, *p*-values were estimated using a Monte Carlo simulation with 10,000 replicates. Logistic regression was not performed because the dataset did not include sufficient patient-level covariates to allow adjustment for potential confounders; the primary aim of this study was exploratory mapping of associations rather than building predictive models. A *p*-value of less than 0.05 was considered statistically significant. A heatmap was created using Microsoft Excel's conditional formatting feature, using a gradient scale to represent data values for visual clarity. The heatmap color scale was applied based on relative values across the entire range of all cells, with higher values shown in red, lower values in green, and intermediate values in shades of yellow. Cells with values less than 10 were excluded from color scaling. Each cell in a heatmap displays both the number of cases and the corresponding percentage; colors indicate only relative magnitude and do not represent statistical significance.

### 2.4 Ethical consideration

This study was approved by the ethical board of FHUH (Approval number HM22–205). Informed consent for each patient was waived and obtained through an opt-out process. Information about the study was made publicly available on the university's website, allowing patients to review the study details and opt out if they wished not to participate.

## 3 Result

### 3.1 Demographic data of prescribing errors

Between April 2019 and March 2020 at FHUH, clinical pharmacists made 9774 pharmaceutical interventions from 3,834,897 drug prescriptions. Of these, 3372 interventions were identified as related to information available in the HIS and were included in this study. The remaining interventions were related to issues such as dosage, duration of use, duplication, or other reasons.

Among the 3372 relevant interventions, the meanpatient age was 63.9 years (SD 20.1), with the 60–80 age group accounting for the highest number of interventions (*n* = 1752, 52.0 %) (Table 1). The distribution of interventions by gender was nearly equal, with 1819 interventions for male patients and 1553 for females. Interventions were more frequent in the Department of Internal Medicine compared to the Surgical Department (2030 vs. 1342). Most interventions occurred on weekdays (*n* = 3027, 89.8 %) with fewer on weekends (Saturday and Sunday) (*n* = 345, 10.2 %). The most frequently involved drug category was anti-tumor agents (*n* = 1200, 33.6 %), followed by antibiotics (*n* = 642, 19.0 %) and anti-inflammatory drugs (*n* = 239, 7.1 %).

**Table 1:** Patient demographics and characteristics of drug prescriptions involved in pharmaceutical interventions.

n		3372
Age, mean (SD)		63.9 (20.1)
Gender, n (%)	female	1553 (46.1)
	male	1819 (53.9)
Department, n (%)	internal medicine	2030 (60.2)
	surgery	1342 (39.8)
Day of week, n (%)	weekday	3027 (89.8)
	weekend	345 (10.2)
Types of drugs, n (%)	anti-tumor agent	1155 (34.3)
	antibiotics	643 (19.1)
	anti-inflammatory drugs	236 (7.0)
	digestive drug	202 (6.0)
	cardiovascular drug	196 (5.8)
	anabolic agent	136 (4.0)
	parenteral nutrition	108 (3.2)
	anti-coagulant drugs	107 (3.2)
	others	589 (17.5)

Data are presented as numbers with percentages or as means with standard deviations.

### 3.2 Patient information in HIS using pharmaceutical intervention

**Fig. 1:**
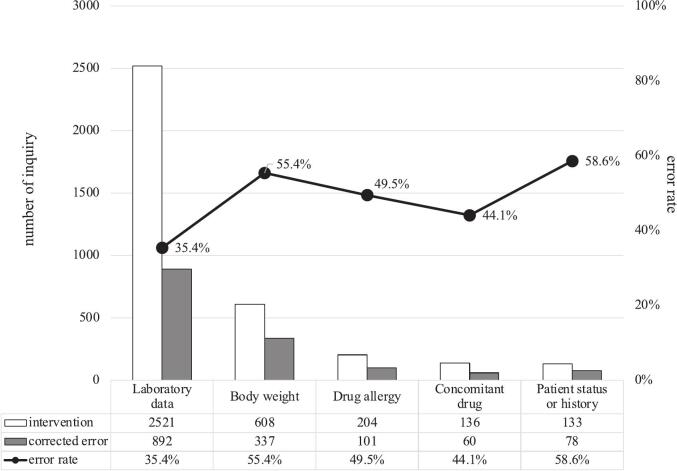
Sources of patient information and associated error rates. Bar graphs show the number of pharmaceutical interventions and the number of corrected errors associated with each patient information source. The error correction rate (corrected errors per intervention) is represented by a line graph.

Laboratory data were the most frequent source of patient information (*n* = 2526), followed by patient weight (*n* = 608), allergy information (*n* = 204), concomitant drugs (*n* = 136), and patient illness or status (*n* = 133) (Fig. 1). Some interventions utilized more than one information source, with 224 involving two sources and 3 involving three. Among laboratory data, the most commonly referenced parameter was renal function (*n* = 1248), followed by complete blood count (*n* = 637), potassium concentration (*n* = 444), liver function (*n* = 151), and calcium concentration (*n* = 92).

### 3.3 High risk area of prescription error detected by pharmaceutical interventions

Of 3372 pharmaceuticalinterventions, 1352 (40.1 %) detected prescribing errors and resulted in corrections. Interventions based on patient status and history had the highest error rate (58.6 %), followed by patient weight (55.4 %), allergy information (49.5 %), and concomitant drugs (44.1 %) (Fig. 1). In contrast, laboratory data had the lowest error rate (35.4 %). There were no significant differences for error rate by gender or department (Table 2). The error rate for weekend interventions was significantly higher than on weekdays; however, the effect size was small (*P* < 0.001, Cramér's V = 0.111), suggesting a weak association. Age also influenced error rates with a medium association (P < 0.001, Cremer's V = 0.207) and exhibited a U-shaped pattern, with younger patients (<20 years: 66.9 %) having the highest rates and older patients (>80 years: 49.9 %) having the second highest rates. Drug type was a significant variable for error rate and suggesting a stronger association (P < 0.001, Cramér's V = 0.395). Interventions for anti-tumor agent and parenteral nutrition were lower error rate (15.9 % and 25.5 %, respectively). In contrast, over 50 % error rates were observed for antibiotics, anti-inflammatory drugs, digestive drugs and anti-coagulants. Among these, digestive drugs had highest rate of prescribing errors, including issues with famotidine and other renal dosing drugs.

**Table 2:** Relationship between pharmaceutical interventions and corrected errors across variables.

variables		corrected error / intervention	error rate (%)	p-value	Cramér's V
Gender	F	638 / 1553	41.1	0.586	0.018
	M	714 / 1819	39.3		
Department	Internal medicine	809 / 2030	39.9	0.147	0.006
	Surgical	543 / 1342	40.5		
Workdays	weekday	1158 / 3027	38.3	<0.001	0.111
	weekend	194 / 345	56.2		
Age	<20	178 / 266	66.9	<0.001	0.207
	20–59	231 / 641	36.0		
	60–79	587 / 1752	33.5		
	>80	356 / 713	49.9		
Drug type	anti-tumor agent	191 / 1200	15.9	<0.001	0.395
	antibiotics	345 / 642	53.7		
	anti-inflammatory drugs	149 / 239	62.3		
	cardiovascular drug	87 / 196	44.4		
	digestive drug	105 / 152	69.1		
	anabolic agent	59 / 137	43.1		
	parenteral nutrition	28 / 110	25.5		
	anti-coagulant drugs	61 / 107	57.0		
	others	327 / 589	55.5		

Data are presented as numbers with percentages. The chi-square test was used for statistical analysis.

### 3.4 Heatmap of high-risk area for prescribing error

**Fig. 2:**
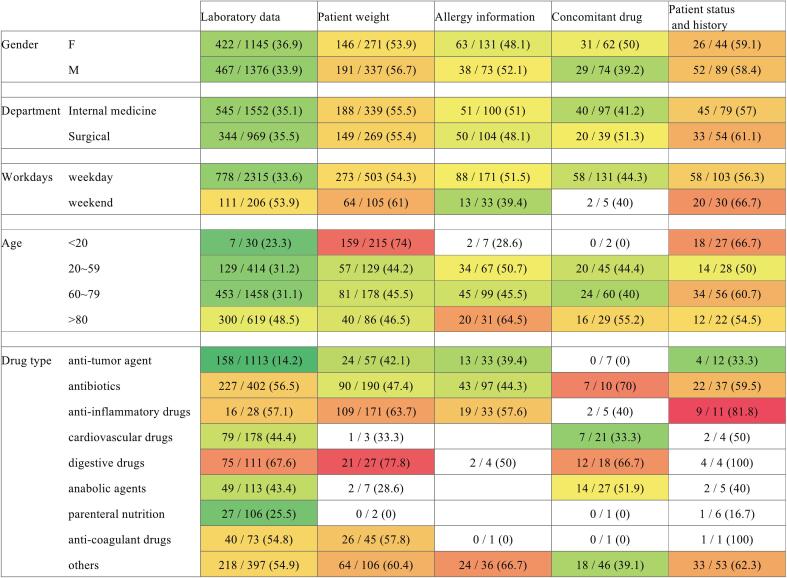
Heatmap of prescription errors detected through pharmaceutical interventions. Data are presented as the number of errors per intervention with corresponding percentages in each cell. Colors indicate relative magnitude only and are not statistically tested. Higher values are shown in red, lower values in green, and intermediate values in yellow. Cells with fewer than 10 cases were excluded from color scaling. (For interpretation of the references to color in this figure legend, the reader is referred to the web version of this article.)

**Fig. 3:**
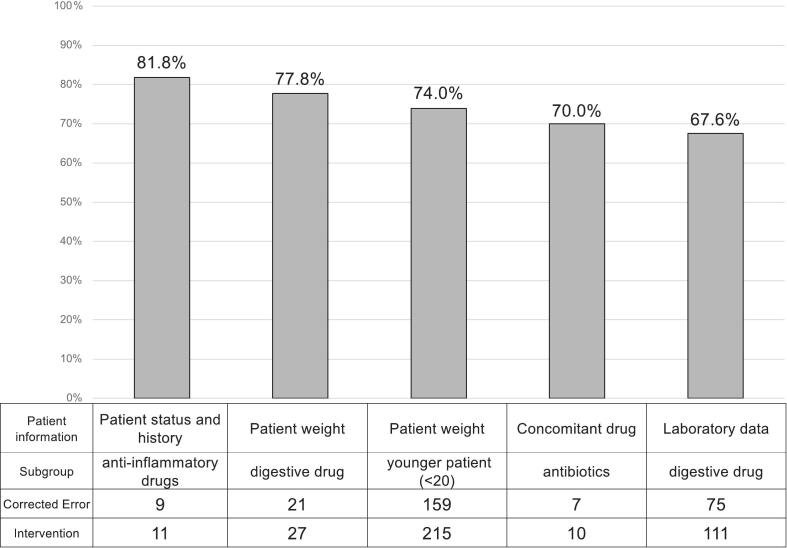
Bar chart of the top five high-risk subgroups identified from the heatmap. The figure shows the five subgroups with the highest prescribing error rates, extracted from the overall heatmap analysis. Subgroups with fewer than 10 interventions were excluded from ranking. Error rates are displayed as bar heights, and the corresponding n/N values are provided in the data table beneath the graph. Subgroups are presented in descending order of error rate. Graphical abstract illustrating the study design and key findings. Laboratory data was the most frequently utilized patient information source, and the overall prescribing error rate was 40.1 %. Three high-risk areas for prescribing errors were identified: weekend prescribing, digestive drugs prescribed based on laboratory data, and younger patients requiring weight-based dosing.

Prescription errors varied depending on prescription variables and information sources. (Fig. 2) Error rates exceeding 70 % were observed in patient weight data for younger patients and digestive drugs, concomitant drugs data for antibiotics, and patient status data for anti-inflammatory drugs. (Fig. 3) Intervention related to patient weight for younger patients primarily involved issues with dosage adjustments or restrictions for pediatric use. Patient weight data with digestive drugs often included anti-emetics for pediatrics and antacids for lower creatinine-clearance, adjusted for patient weight. Patient status data with anti-inflammatory drugs included cases of NSAIDs for pregnant patients, pediatric patients, and patients with a history of asthma. Concomitant drugs data with antibiotics highlighted drug-drug interactions, such as those involving fluconazole and clarithromycin, as well as other antibiotics. In contrast, anti-tumor agents with laboratory data had the lowest error rate (14.2 %) within the heatmap. These interventions included laboratory abnormalities, such as verification of white cell count for determining indications for chemotherapy, renal function for dosage adjustments, and blood test results for mitigating adverse effects of chemotherapy. Stratified analyses were performed to supplement the descriptive heatmap findings. A weekend effect was significant only for errors related to laboratory data. Age was significantly associated with errors in both laboratory data and patient weight. Drug type was also significantly associated with errors in laboratory data and patient weight, but not with other categories. The results of these stratified analyses are provided in Supplementary Table 1.

## 4 Discussion

This study investigated prescribing errors detected through pharmaceutical interventions that had bypassed the information-checking system, with a focus on the type of patient information involved. Among the 9774 pharmaceutical interventions, more than one-third of these were directly related to patient information already stored in the HIS, representing avoidable errors caused by limitations in the checking system. Notably, 40.1 % of these interventions resulted in corrections, thereby preventing potential medication errors. High-risk areas identified included weekend settings and younger patients. The heatmap analysis further highlighted that weight data for younger patients was a particularly vulnerable area for prescribing errors. Previous studies have highlighted the critical role of laboratory data, including renal function, in ensuring safe prescribing.^18^ However, few studies have quantitatively examined the association between laboratory data utilization and prescribing errors.^33^ Our findings provide empirical evidence that supports the importance of effective laboratory data management in preventing medication errors.

The most frequently used information for interventions in this study was laboratory data. Previous studies have highlighted the importance and frequent misuse of laboratory data, including renal functions, liver enzymes, and blood cell counts, as common source of prescribing errors in clinical settings, which is consistent with the findings of this study.^17^^,^^18^^,^^34^ Many drugs require adjustments based on laboratory data for dosage, suspension, or substitution, necessitating physician to review these data for each prescription.^6^, ^7^, ^8^, ^9^ Although physicians have access to health records through the HIS, their workload associated with retrieving laboratory data may reduce their utilization, contributing to medication errors. Since current alert systems are insufficient to support decision-making, a simple strategy to enhance laboratory data utilization, such as displaying the latest test results prominently, could be a viable option to reduce prescribing errors.^33^ Real-time integration of laboratory data into dose adjustment at the point of prescribing could also reduce prescribing errors. Further research is warranted to test the effectiveness of real-time laboratory data display and integration on reducing prescribing errors and drug adverse events.

Our study identified variations in prescribing errors related to weekends and ages. This trend may be attributed to weekend physician rotations or non-pediatricians prescribing for pediatric patients. On weekends, the emergency room (ER) is staffed with junior physicians under a rotation system, often without attending physicians or managers. Staffing shortages may also reduce opportunities for cross-checking, while workload fluctuations and weekend-specific patient backgrounds could further increase error rates. In ER settings, medication-related errors are especially likely from a human factors' perspective. Physicians are frequently exposed to interruptions and simultaneous patient management, which require multitasking and result in a high cognitive load, all of which can increase the likelihood of errors.^35^, ^36^, ^37^ Previous studies support the idea that prescribing processes vary based on clinical circumstances, such as the day of the week or shift hours, with errors being more common during these periods.^38^ Our findings are consistent with this observation. Many prescriptions for pediatric patients required physicians to adjust dosing based on age and body weight, which differed from prescribing for adult patients. Current checking systems generate alerts based on predefined rules but fail to account for such high-risk clinical situations.^39^ Targeted approaches specifically for weekend settings and prescriptions by non-pediatricians for younger patients could help mitigate alert fatigue. Since weight-based dosing errors in pediatric patients, which we observed, have also been reported in earlier studies, practical countermeasure may help reduce such errors.^14^^,^^18^ These include the development of standardized dosing tables for frequently used pediatric drugs, and incorporating weight-based dosing support into the prescribing screen by providing a default calculated dose (body weight × dosage) that physicians can adjust if necessary.^40^ Such measures could assist non-pediatricians in preventing prescribing errors while minimizing additional workload. This would prevent errors proactively and reduce risks without adding extra steps for prescribers. Further research is needed to explore these high-risk contexts, particularly weekend prescribing and pediatric dosing, to inform targeted preventive strategies. In addition to technical system improvements, our findings have implications for training and policy. For example, physician and pharmacist training curricula could place greater emphasis on pediatric weight-based dosing and renal dose adjustment, which were identified as high-risk areas. At the hospital level, policies could include requiring non-pediatricians to consult standardized pediatric dosing tables when prescribing for children, and requiring pharmacists to contact the prescriber regarding pediatric prescriptions regardless of whether the calculated dose appears appropriate.

Anti-tumor agents were intensively monitored to ensure the safety of chemotherapy processes, resulting in a higher number of interventions but a lower error rate. Previous studies have reported that approximately 30–40 % or more of chemotherapy-related prescriptions requiring pharmaceutical interventions were corrected,^41^, ^42^, ^43^ whereas our results showed a lower correction rate of 14.9 %. This difference may be because our study focused only on information-checking errors detected by pharmacists through the HIS. Interventions, such as pre-prescribing discussions between pharmacists and physicians, were not included. Another contributing factor to the high frequency and low error rate was the intensive and redundant checking processes for chemotherapy, which functioned as a fail-safe strategy. Many interventions addressed minor deviations from laboratory data limits, reflecting the stringent safety oversight by pharmacists. The appropriate error rates in these redundant strategies have not been established; further studies are necessary to investigate the standard criteria for pharmaceutical interventions.

Digestive drugs s had a high error rate, most frequently involving renal dose adjustments for famotidine. Previous studies have identified renal dosing drugs including famotidine, as a high-risk area for medication errors, highlighting the challenges of adjusting renal dosing.^44^^,^^45^ Strategies developed in community pharmacies have improved renal dose adjustment but may not be directly applicable in acute hospital settings.^46^ Delegating renal dosing decisions from physicians to pharmacists could be an alternative countermeasure. Further investigation is needed to determine which types of system assistance or implementation processes would make electronic systems more effective in this area.

This study has several limitations. It was conducted in a single academic institution in a high-income country, which may limit the generalizability of the findings to other institutions, healthcare systems, or countries with different regulations, different information systems or prescribing verification processes. Although advanced CPOE systems were implemented in this study, the implications of the identified high-risk areas—such as pediatrics, weekend prescribing, and renal dosing—may have international relevance across healthcare systems in both low-, middle- and high-income countries. For example, standardized pediatric dosing tables and simple renal dosing checklists could serve as practical safeguards even in the absence of electronic prescribing systems. The institutional context, including physician and pharmacist experience, patient demographics, and clinical workload, which may influence error rates, could not be investigated in this study. However, some of the findings may be applicable to other healthcare settings, such as dosing error in weekend for pediatric patients could be community hospitals with physician rotation system or underutilization of laboratory data could be both other acute care hospitals or outpatient clinics, particularly those with similar prescribing systems and workflows. Second limitation is that this study did not assess pre-prescribing interventions involving discussions between pharmacists and physicians, nor did it evaluate patient outcomes related to medication errors. There remains a risk of underestimation of prescribing errors and pharmaceutical interventions, as approximately 20 % of pharmaceutical interventions involved the addition of drugs, which were not included in this study because these interventions were performed before physicians' orders.^47^ Although pharmacist interventions likely prevented many harmful errors, the corrections recorded in this study serve only as surrogate measures of error prevention. Finally, this study did not aim to identify independent risk factors, and multivariable analyses such as logistic regression were not feasible because of the wide variety of drugs and the limited availability of patient-level covariates. As a result, potential confounding factors could not be adjusted for, and our findings should be interpreted as descriptive associations rather than independent risk factors. Nevertheless, we believe our findings remain informative for identifying high-risk situations in prescribing and guiding future safety strategies.

## 5 Conclusion

This study highlights the significance of five key patient information sources in preventing prescribing errors. Laboratory data were most frequently used, suggesting that improving their accessibility within information systems could help prevent errors. The risk heatmap identified weekends, pediatric patients, and renal dosing as high-risk areas, highlighting the interaction between patient information and prescribed drugs. These findings may guide future system improvements, such as context-aware alerts, prioritizing checks in high-risk settings, enhancing laboratory data display, or integrating automated weight-based dosing calculators. These findings may inform future system improvements, such as context-aware alerts, prioritizing checks in high-risk settings, enhancing laboratory data display, or integrating automated weight-based dosing calculators. Beyond AI-driven error detection, system design should also focus on proactive error prevention at the point of order entry. Further studies across diverse healthcare environments are needed to validate and expand upon these results.

## CRediT authorship contribution statement

**Daisuke Koike:** Writing – original draft, Data curation, Conceptualization. **Masahiro Ito:** Writing – review & editing, Supervision. **Keiko Tomomatsu:** Writing – review & editing, Data curation. **Ryuta Shindo:** Writing – review & editing, Conceptualization. **Terumi Miyashita:** Writing – review & editing, Conceptualization. **Junichi Yamakami:** Writing – review & editing, Conceptualization. **Akihiko Horiguchi:** Writing – review & editing, Supervision. **Shigeki Yamada:** Writing – review & editing, Data curation.

## Funding

This research did not receive any specific grant from funding agencies in the public, commercial, or not-for- profit sectors.

## Declaration of competing interest

The authors declare that they have no competing interests.

## Appendix A Supplementary data

Supplementary material Image 1
