# Supplementary material for: The impact of patient information on prescribing errors: Insights from pharmaceutical interventions
Source: Explor Res Clin Soc Pharm. 2025 Oct 6;20:100665. doi: 10.1016/j.rcsop.2025.100665 (PMC12547715; doi:10.1016/j.rcsop.2025.100665)
Supplement: Supplementary file 1 — Supplementary material [file mmc1.docx]

|  | Laboratory data | | | Patient weight | | | Allergy information | | | Concomitant drug | | | Patient status and history | | |
| --- | --- | --- | --- | --- | --- | --- | --- | --- | --- | --- | --- | --- | --- | --- | --- |
|  | Error / Intervention (n) | Error rate (%) | p-value | Error / Intervention (n) | Error rate (%) | p-value | Error / Intervention (n) | Error rate (%) | p-value | Error / Intervention (n) | Error rate (%) | p-value | Error / Intervention (n) | Error rate (%) | p-value |
| Gender |  |  | 0.132 |  |  | 0.512 |  |  | 0.662 |  |  | 0.228 |  |  | 1 |
| F | 422 / 1145 | 36.9 |  | 146 / 271 | 53.9 |  | 63 / 131 | 48.1 |  | 31 / 62 | 50 |  | 26 / 44 | 59.1 |  |
| M | 467 / 1376 | 33.9 |  | 191 / 337 | 56.7 |  | 38 / 73 | 52.1 |  | 29 / 74 | 39.2 |  | 52 / 89 | 58.4 |  |
|  |  |  |  |  |  |  |  |  |  |  |  |  |  |  |  |
| Department |  |  | 0.864 |  |  | 1 |  |  | 0.779 |  |  | 0.341 |  |  | 0.721 |
| Internal medicine | 545 / 1552 | 35.1 |  | 188 / 339 | 55.5 |  | 51 / 100 | 51 |  | 40 / 97 | 41.2 |  | 45 / 79 | 57 |  |
| Surgical | 344 / 969 | 35.5 |  | 149 / 269 | 55.4 |  | 50 / 104 | 48.1 |  | 20 / 39 | 51.3 |  | 33 / 54 | 61.1 |  |
|  |  |  |  |  |  |  |  |  |  |  |  |  |  |  |  |
| Workdays |  |  | <0.001* |  |  | 0.236 |  |  | 0.255 |  |  | 1 |  |  | 0.4 |
| weekday | 778 / 2315 | 33.6 |  | 273 / 503 | 54.3 |  | 88 / 171 | 51.5 |  | 58 / 131 | 44.3 |  | 58 / 103 | 56.3 |  |
| weekend | 111 / 206 | 53.9 |  | 64 / 105 | 61 |  | 13 / 33 | 39.4 |  | 2 / 5 | 40 |  | 20 / 30 | 66.7 |  |
|  |  |  |  |  |  |  |  |  |  |  |  |  |  |  |  |
| Age |  |  | <0.001* |  |  | <0.001* |  |  | 0.198 |  |  | 0.386 |  |  | 0.611 |
| <20 | 7 / 30 | 23.3 |  | 159 / 215 | 74 |  | 2 / 7 | 28.6 |  | 0 / 2 | 0 |  | 18 / 27 | 66.7 |  |
| 20~59 | 129 / 414 | 31.2 |  | 57 / 129 | 44.2 |  | 34 / 67 | 50.7 |  | 20 / 45 | 44.4 |  | 14 / 28 | 50 |  |
| 60~79 | 453 / 1458 | 31.1 |  | 81 / 178 | 45.5 |  | 45 / 99 | 45.5 |  | 24 / 60 | 40 |  | 34 / 56 | 60.7 |  |
| >80 | 300 / 619 | 48.5 |  | 40 / 86 | 46.5 |  | 20 / 31 | 64.5 |  | 16 / 29 | 55.2 |  | 12 / 22 | 54.5 |  |
|  |  |  |  |  |  |  |  |  |  |  |  |  |  |  |  |
| Drug type |  |  | <0.001* |  |  | <0.001* |  |  | 0.1034 |  |  | 0.1003 |  |  | 0.05619 |
| anti-tumor agent | 158 / 1113 | 14.2 |  | 24 / 57 | 42.1 |  | 13 / 33 | 39.4 |  | 0 / 7 | 0 |  | 4 / 12 | 33.3 |  |
| antibiotics | 227 / 402 | 56.5 |  | 90 / 190 | 47.4 |  | 43 / 97 | 44.3 |  | 7 / 10 | 70 |  | 22 / 37 | 59.5 |  |
| anti-inflammatory drugs | 16 / 28 | 57.1 |  | 109 / 171 | 63.7 |  | 19 / 33 | 57.6 |  | 2 / 5 | 40 |  | 9 / 11 | 81.8 |  |
| cardiovascular drug | 79 / 178 | 44.4 |  | 1 / 3 | 33.3 |  | 0 / 0 |  |  | 7 / 21 | 33.3 |  | 2 / 4 | 50 |  |
| digestive drug | 75 / 111 | 67.6 |  | 21 / 27 | 77.8 |  | 2 / 4 | 50 |  | 12 / 18 | 66.7 |  | 4 / 4 | 100 |  |
| anabolic agent | 49 / 113 | 43.4 |  | 2 / 7 | 28.6 |  | 0 / 0 |  |  | 14 / 27 | 51.9 |  | 2 / 5 | 40 |  |
| parenteral nutrition | 27 / 106 | 25.5 |  | 0 / 2 | 0 |  | 0 / 0 |  |  | 0 / 1 | 0 |  | 1 / 6 | 16.7 |  |
| anti-coagulant drugs | 40 / 73 | 54.8 |  | 26 / 45 | 57.8 |  | 0 / 1 | 0 |  | 0 / 1 | 0 |  | 1 / 1 | 100 |  |
| others | 218 / 397 | 54.9 |  | 64 / 106 | 60.4 |  | 24 / 36 | 66.7 |  | 18 / 46 | 39.1 |  | 33 / 53 | 62.3 |  |

Supplementary Table 1. Stratified chi-square and Fisher’s exact test results for patient information categories, supplementing the heatmap findings. Data are presented as numbers with percentages. P-value calculated using Fisher’s exact test; Monte Carlo simulation with 10,000 replicates was applied where exact computation was infeasible.
